# Perioperative Management of a Needle-Phobic Parturient for a Cesarean Section: A Case Report

**DOI:** 10.7759/cureus.59032

**Published:** 2024-04-25

**Authors:** Norihiko Obata, Eri Nakai, Shohei Makino, Satoshi Mizobuchi

**Affiliations:** 1 Department of Anesthesiology, Kobe University Hospital, Kobe, JPN; 2 Department of Anesthesiology, Hyogo Prefectural Kobe Children's Hospital, Kobe, JPN

**Keywords:** general anesthesia, perioperative management, intensive care unit, cesarean section, needle phobia

## Abstract

We describe the perioperative management of a pregnant woman with severe needle phobia who underwent a cesarean section. General anesthesia with slow induction using an inhalant anesthetic for cesarean section is a rare and unique situation. Furthermore, the management of this case was more complicated because the patient not only refused the puncture procedure but also refused the presence of an indwelling object when she woke up from the anesthesia. After the operation, the patient was admitted to the intensive care unit (ICU) and received mechanical ventilation under deep sedation. The patient was managed under sedation until the day after surgery, and both mother and child progressed without perioperative complications.

## Introduction

Needle phobia, most of which is based on painful past experiences, is highly prevalent in varying degrees [1]. Needle phobia interferes with medical practice in a variety of settings, especially in painful procedures [2,3]. On the other hand, it is widely known that pregnant women are at high risk of aspiration, and rapid sequence induction is indicated for general anesthesia [4]. Hence, general anesthesia management using slow induction for cesarean section is an extremely unique situation.

In this report, we describe the perioperative management of a pregnant woman with severe needle phobia who underwent a cesarean section under general anesthesia.

This article was previously presented as a meeting abstract at the 125th Annual Meeting of the Japan Society for Obstetric Anesthesia and Perinatology in 2021.

## Case presentation

The patient was a 27-year-old pregnant woman who had been abused by her mother since childhood. She had refused all medical puncture procedures since she received an intramuscular injection when she was involuntarily admitted to a psychiatric hospital at the age of 16.

The patient was referred to the Department of Obstetrics and Gynecology at our hospital at 14 weeks gestation, but she refused to have a blood test with a puncture. She also refused to have her veins secured while awake, to have regional anesthesia, or to have catheters in place while awake. Because of the presence of placenta previa, the obstetrician consulted us about perioperative management of the patient in the event of a cesarean section, and we discussed the ethical aspects of this procedure in the hospital. As a result, it was decided to continue the delivery management at our hospital after explaining to the patient the risks of delivery management without blood collection tests and intravenous line securement and obtaining consent.

**Figure 1:**
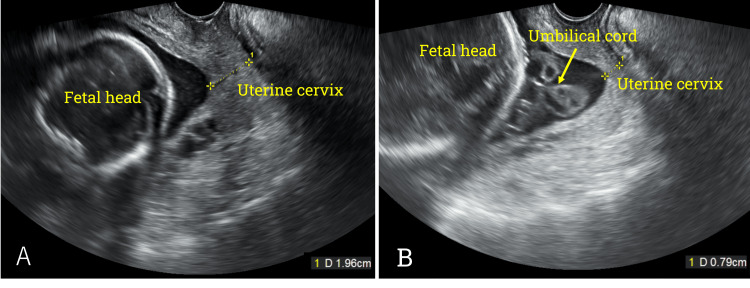
Transvaginal ultrasound findings A: At 26 weeks gestation, the cervical length was 1.96 cm. B: At 33 weeks gestation, a forelying umbilical cord was revealed between the fetal head and the uterine cervix (yellow arrow). The cervical length was shortened to 0.79 cm.

At 33 weeks and five days of pregnancy, the patient was admitted to the hospital for placenta previa and a forelying umbilical cord (Figure 1). An elective cesarean section was scheduled on the second day of hospitalization. Preoperative examinations included an electrocardiogram, chest X-ray, and urinalysis, but the patient refused to have a blood draw with a needle puncture.

For anesthesia, we chose general anesthesia with slow induction using an inhalational anesthetic. Fasting from solid food for six to eight hours and from clear water for two hours before surgery was strictly observed. Under standard monitoring, slow induction was performed with oxygen, 50% nitrous oxide, and 1% sevoflurane. While promoting deep breathing, the inhaled sevoflurane concentration was increased to 3%, and the patient fell asleep gently. After she fell asleep, a peripheral venous line was secured immediately. Then, 200 mcg fentanyl and 70 mg rocuronium were administered intravenously, and the patient was intubated successfully 90 seconds later without positive pressure ventilation. The neonate was delivered 14 minutes after the start of surgery. After fetal delivery, we ceased inhalational anesthesia and transitioned to total intravenous anesthesia using propofol. There was not much bleeding after the delivery of the placenta. The operation time was 84 minutes, and the anesthesia time was 116 minutes. The neonate had an Apgar score of 1 at one minute and 6 at five minutes after birth, and the infant was intubated in the operating room.

After the operation, the patient was managed in the ICU with tracheal intubation under sedation. In the ICU, sedation was continued by continuous administration of propofol (5 mg･kg^-1^･h^-1^), dexmedetomidine (0.5 mcg･kg^-1^･h^-1^), and fentanyl (1 mcg･kg^-1^･h^-1^) under mechanical ventilation. On the first postoperative day, the subcutaneous drain was removed, and the sedative medication was reduced until spontaneous breathing became stable (propofol 3 mg･kg^-1^･h^-1^, dexmedetomidine 0.6 mcg･kg^-1^･h^-1^, fentanyl 1 mcg･kg^-1^･h^-1^). The patient was extubated under sedation after the removal of all indwelling devices except for one venous tract. The patient's respiratory status was then stable, and the last venous tract was removed five minutes after extubation. Her progress was good, and she was discharged on the fifth postoperative day.

## Discussion

We undertook the perioperative management of a pregnant woman with needle phobia for a cesarean section. The patient refused to be punctured, so general anesthesia was administered by slow induction with an inhaled anesthetic without securing the intravenous route. Although there have been past reports of general anesthesia with slow induction for cesarean section [5,6] and there were some reports of anesthesia for needle-phobic patients [7,8], the perioperative management of this case was more complicated because the patient not only refused the puncture procedure but also refused the presence of an indwelling object when she woke up from the anesthesia.

In this case, with a strict restriction of fasting and drinking periods and using 1% to 3% sevoflurane, we were able to safely induce general anesthesia in the parturient without an intravenous route without aspiration. To prevent intraoperative awareness, we aimed for a sufficiently deep level of anesthesia. The neonate was born in a sleeping state 30 minutes after the start of anesthesia, and the infant was intubated. After fetal delivery, to mitigate uterine relaxation, we discontinued the administration of inhalational anesthetics and opted for total intravenous anesthesia using propofol for the subsequent maintenance of anesthesia. This report concerns a severely needle-phobic pregnant woman. However, the anesthesia technique used in this case is potentially applicable to cesarean sections where intravenous access cannot be secured due to reasons such as severe obesity. Additionally, we believe it can be adapted for emergency surgeries, as demonstrated in the reports by Schaut et al. and Que et al. [5,6].

Postoperatively, the patient was moved to the ICU under deep sedation with propofol and other medications for mechanical ventilation. This was because an overnight observation period was necessary to determine that there were no postoperative bleeding complications. On the first postoperative day, after we confirmed that there was no postoperative bleeding, the trachea was extubated under sedation, the sedative medication was finished, and the peripheral venous catheter was removed. When we asked the patient after she fully awakened, she had no recollection of any puncture procedure or indwelling objects and was very relieved. We considered that sedation with propofol and dexmedetomidine and analgesia with dexmedetomidine and fentanyl were very efficacious choices.

As Parrott et al. stated in a similar case report [8], it is sometimes difficult to make a decision when the patient wishes to have a procedure that is perceived as more dangerous by the medical staff, due to ethical issues involved. The critical difference between our case and Parrott's report is that the patient refused to even have the indwelling object in the awake state. We discussed the treatment plan with several obstetricians and anesthesiologists in advance. The risks were fully explained to the patient several times, and consent, including a disclaimer, was obtained.

## Conclusions

We experienced perioperative management of a parturient with needle phobia, who refused the presence of an indwelling object for a cesarean section. Postoperatively, the patient was managed in the ICU and successfully extubated under sedation. Through collaboration with obstetricians, neonatologists, and intensivists, both mother and child could be managed safely without complications.
